# An Initial Scoping Review of Dysregulation of Mood, Energy, and Social Rhythms Syndrome (DYMERS) Regarding Burnout in Healthcare Professionals During COVID-19

**DOI:** 10.3390/jcm14031035

**Published:** 2025-02-06

**Authors:** Carol Nash

**Affiliations:** History of Medicine Program, Department of Psychiatry, Temerty Faculty of Medicine, University of Toronto, Toronto, ON M5S 1A1, Canada; carol.nash@utoronto.ca

**Keywords:** dysregulation, biological rhythms, social rhythms, behavioral rhythms, DYMERS, healthcare professionals, COVID-19, burnout

## Abstract

**Background/Objectives**: Dysregulation of Mood, Energy, and Social Rhythms Syndrome (DYMERS) characterizes the poor regulation of biological (sleep/waking), social, and behavioral rhythms that affected the level of burnout in healthcare professionals during the pandemic in particular. The aim is to provide an initial scoping review of publications on this topic. **Methods**: The keywords “Stress Rhythms Dysregulation Bipolar Disorder Burnout DYMERS Healthcare professionals COVID-19” were searched on 9 December 2024 following PRISMA 2020 guidelines, using five primary databases (OVID, ProQuest, PubMed, Scopus, Web of Science), one register (Cochrane COVID-19 register), and one supplementary database (Google Scholar). Included were peer-reviewed publications. Excluded were duplicates, reports lacking either a research study or any keywords, or including irrelevant information regarding them. **Results**: The returns for all the databases were (n = 0) except for ProQuest (n = 4) and Google Scholar (n = 14). Of these, three ProQuest returns were duplicates of the Google Scholar search. The remaining report contained irrelevant information on healthcare professionals. The Google Scholar search results produced two relevant reports—neither duplicated with ProQuest. The excluded contained a duplicate in the search itself, three that did not mention healthcare professionals, two that contained irrelevant information concerning them, four returns that were not a research study, and three that were not peer-reviewed. **Conclusions**: The two studies published on this topic are by various members of the same investigating institution. DYMERS has provided valuable insights regarding burnout in healthcare professionals. The suggestion is for further DYMERS research by this team and others, anticipating future pandemics.

## 1. Introduction

In contrast to the American Psychiatric Association, DSM-5, separation of bipolar disorders from depressive disorders [1], the neo-Kraepelinian concept of a spectrum of mood disorders, including bipolar disorders and depressive disorders, is gaining attention [2]. This notice results from some clinical settings tools, particularly the Mood Disorder Questionnaire (MDQ), identifying more false positives compared to diagnoses of bipolar disorder [3] and the establishment of the disorders observed among the false positives in the previous decade [4,5] and again recently [6] as having a strong association with bipolar disorder. No link has been identified between the genetic risk of bipolar disorder and MDQ scores [7]. However, the genetics of bipolar disorder involve multiple interactive risk factors [8]. Consequently, over the last few years, in response to the COVID-19 pandemic of 2020–2023 [9,10], a “heuristic” hypothesis emerged that MDQ positivity could identify a “Dysregulation of Mood, Energy, and Social Rhythms Syndrome” (DYMERS) with a crucial role in the worsening of chronic conditions related to stress, and social rhythms in stress prevention [11,12], particularly regarding burnout in healthcare professionals. Although the MDQ is not an instrument that identifies DYMERS [13], there was a serendipitous discovery that MDQ-positive patients had elements of DYMERS, as the MDQ measures a dimension shared with other conditions than bipolar disorder [14]. The authors did not identify DYMERS as a typical burnout syndrome of professionals, but rather as a typical chronic stress syndrome of which the burnout of healthcare professionals (for example, during the pandemic) is an example.

The history of DYMERS began when dysregulation regarding biological rhythms and social patterns in association with bipolar disorder was evidenced in a large population-based study in 2017 [15]. This research then was informative to a 2020 publication in demonstrating that biological rhythms are a contributing factor in the pathophysiology of mood disorders, finding increased inflammatory biomarkers connected to changes in biological rhythms in bipolar disorder [16]. It was later that same year, in December, that another publication sought to understand bipolar disorder within a biopsychosocial emotion dysregulation framework directly [17] following an earlier systematic review published in July 2020 linking dysregulation to burnout [18]. A 2022 publication investigated sleep disturbances in the context of neurohormonal dysregulation in patients with bipolar disorder [19]. However, intensive research on this topic began regarding burnout and COVID-19 in studies undertaken initially by the Carta and Ouali research team concerning the lockdown in Tunis, Tunisia [12], expanding to include additional researchers at the University of Cagliari, Cagliari, Italy [11], particularly Primavera, with several publications, such as two in 2023 [20,21] and two in 2024 [22,23].

In an editorial as part of this Special Issue by Carta, Karan, and Cossu [23] regarding stress, dysregulation of rhythms, and bipolar disorder, the authors note that one group was under significant pressure during COVID-19 and had more dysregulation of personal and social rhythms, and to be at greater risk for burnout syndrome and mood disorders than others—healthcare workers. An assessment based on four studies was conducted during the pandemic regarding healthcare providers [24,25,26,27], the first three of which regarded the epicenter of the pandemic in Italy—a country with high levels of COVID-19 mortality [28,29]. Most of these healthcare workers studied were healthcare professionals, specifically physicians and nurses.

Described first in 1974 [30], the World Health Organization defines burnout as an occupation-dependent syndrome from unsuccessfully managed chronic workplace stress characterized by energy depletion or exhaustion and an increased work-related mental distance, negativism, or cynicism, reducing professional efficacy [31]. Burnout is a significant cause of healthcare professional turnover [32], producing increased on-the-job errors and reduced patient care [33]. Before COVID-19, burnout was widespread in healthcare professionals, with over one-half of physicians and one-third of nurses experiencing symptoms in the US [34]. Ending as a global health emergency on 5 May 2023 [35], COVID-19 has continued as a factor in escalating healthcare professional burnout since the beginning of the pandemic [36] on 11 March 2020 [37]—further increasing the complexity of solutions to burnout in healthcare professionals [38,39].

The psychosocial implications of burnout in healthcare professionals remain a conundrum [40]—especially regarding the effects of COVID-19 in increasing the complexity of their burnout [41]. Consequently, what is intriguing and novel regarding the diagnosis of DYMERS along with their burnout is that the identification of disrupted psychosocial rhythms comes to the forefront. For example, disruptions in circadian rhythms and poor sleep quality and quantity significantly impacted the long-term mental health of nurses working night shifts during COVID-19 [42].

Based on this identification of healthcare professionals as likely to have been more affected by DYMERS and burnout during COVID-19, and because burnout is an enduring problem in healthcare providers augmented as a result of COVID-19, this scoping review is undertaken to investigate the range of publications that have studied this phenomenon. Consequently, this is not a literature review on burnout in all healthcare workers during COVID-19. The attention is to the extent of research published on this novel finding of DYMERS regarding healthcare professionals resulting from the pandemic.

This scoping review represents the first undertaken on this novel topic, making the results, though initial, significant.

## 2. Materials and Methods

The gathered materials and methods used regard the identification of studies through searches of databases and registers following PRISMA guidelines for scoping reviews [43,44]. The PRISMA process for scoping reviews has been internationally standardized [45] and is now considered best practice guidance for scoping reviews [46]. The method includes a selection of databases and registers in searching the keywords, then removing each of (1) the duplicates, (2) ineligible records marked by the automation tools, (3) records not in English, and (4) those not peer-reviewed. The records screened exclude those lacking healthcare professionals first, as this parameter represents the most differentiated keyword. Those not containing healthcare professionals may also not contain DYMERS, burnout, or COVID-19. However, no further consideration was given to these same reports once eliminated because of missing healthcare providers. There was an assessment of eligibility, excluding those lacking relevant keyword information. This process resulted in the studies included in the review—all of which represented the reports of the included studies.

**Figure 1:**
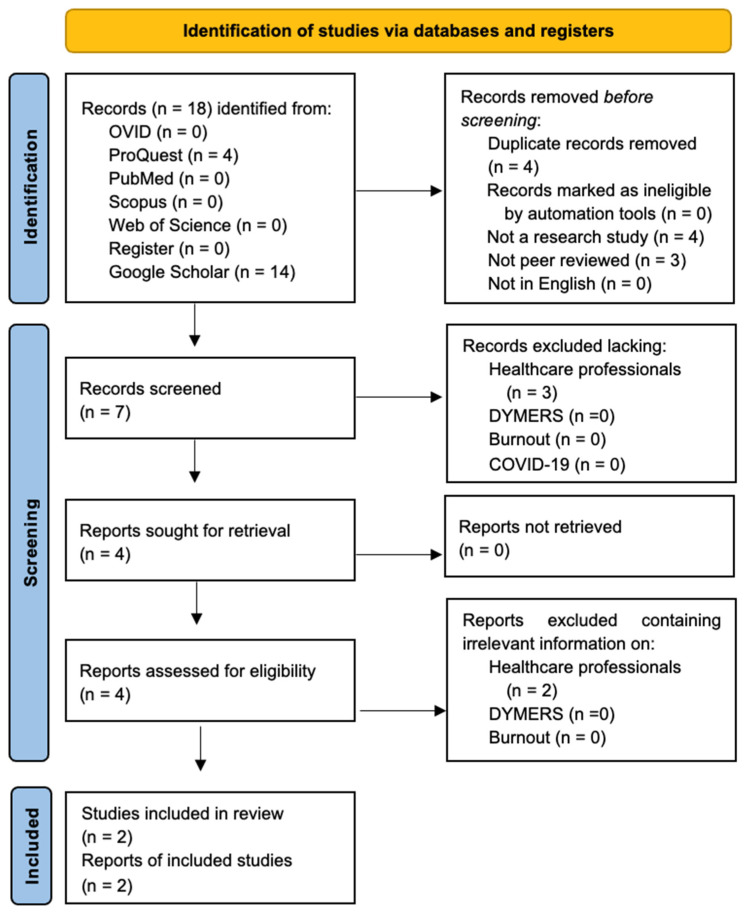
PRISMA flow diagram of results of December 2024 scoping review of “Stress Rhythms Dysregulation Bipolar Disorder Burnout DYMERS Healthcare Professionals COVID-19” of primary databases OVID, ProQuest, PubMed, Scopus, and Web of Science, Register Cochrane COVID-19 register, and Supplementary Database Google Scholar, based on PRISMA requirements [43].

The result is a preferred reporting item for systematic review and meta-analyses (PRISMA) flow of information diagram specific to scoping reviews. The most recent PRISMA template for scoping reviews [47] is the basis of Figure 1 in the Section 3. Figure 1 follows the 9 December 2024 exclusion and inclusion criteria flow. The PRISMA Scoping Review Checklist is in a supplementary file (Supplementary S1: Preferred Reporting Items for Systematic Reviews and Meta-Analyses Extension for Scoping Reviews (PRISMA-ScR) Checklist) outlining the process undertaken in this article. The process pre-registration is at OSF Registries: https://osf.io/c4bv2 (accessed on 1 January 2025).

The selection of a scoping review for this search was to find the range and depth of research on this subject rather than to examine PICO (population, intervention, comparison, and outcome), requiring a systematic review [48]. The scoping review searches five primary databases (OVID, ProQuest, PubMed, Scopus, and Web of Science), one register (the Cochrane COVID-19 Register), and one supplementary database (Google Scholar) selected for their relevance and reach [49]. The selection of these databases is those most relevant to healthcare-related topics and most likely to produce the broadest reach [49]. The keywords searched are “Stress Rhythms Dysregulation Bipolar Disorder Burnout DYMERS Healthcare Professionals COVID-19” for each database. Following PRISMA guidelines [45,46,50], those included peer-reviewed, English language, and research studies. Excluded are reports of irrelevant information on any keywords (including those that mention the keywords in the references alone).

As a review concerning COVID-19 investigations, the International Prospective Register of Systematic Reviews (PROSPERO) requirements are relevant. According to the PROSPERO website, “PROSPERO accepts registrations for systematic reviews, rapid reviews, and umbrella reviews. PROSPERO does not accept scoping reviews or literature scans” [51]. In meeting the definition of a scoping review, this review is not a systematic review. In contrast to a scoping review, a systematic review is “A review that uses explicit, systematic methods to collate and synthesize findings of studies that address a clearly formulated question” [52]. As a scoping rather than a systematic review, following the instructions provided, registration with PROSPERO is not permitted.

As per the requirements of the PRISMA flow diagram [43], the databases searched are differentiated only regarding the location of the records. At this point in the process, there are 18 returns—4 from ProQuest and 14 from Google Scholar. All records returned from each database are combined once the “Records removed before screening” is undertaken. As a result, the duplicate records removed in total were (n = 4). There were no records marked as ineligible by automation tools. Those that were not a research study (n = 4), not peer-reviewed (n = 3), and those not in English (n = 0). This process left seven records screened. Of these, the records excluded were no healthcare professionals (n = 3), leaving four reports sought for retrieval with no reports not retrieved. Reports were excluded by reading the articles and finding irrelevant information concerning healthcare professionals (n = 2) regarding the four reports assessed for eligibility. Two studies were left included, totaling two reports of included studies. However, this additive requirement for PRISMA reporting means flow diagrams do not reveal the details of the individual searches..

Google Scholar is considered a supplementary database. It was selected for the search as a 2019 study of twelve academic databases found it the most comprehensive academic search engine [53], additionally reconfirmed with 2023 research [54]. However, in 2020 [49], Google Scholar was evaluated as unsuitable for primary review searches, considering it a supplementary source of evidence. The reason noted is that Google Search delivers inconsistent results. On the other hand, this same 2020 review continued to acknowledge Google Scholar as the most comprehensive database used mainly by academics, regardless of its low precision and lack of support for many of the features of systematic searches. As this is a scoping review and comprehensiveness is key, Google Scholar is significant to the intended purpose of the undertaking.

Of the five primary databases searched, the one register, and the one supplementary database, only ProQuest (as a primary database) and Google Scholar (as a supplementary database) produced returns. Of the returned results, the first four entries are from the ProQuest search, and the last 14 are the result of the Google Scholar search. Of note is that three of the four returns from the ProQuest search are duplicates in the Google Scholar search, and the only returns in the ProQuest search were publications from 2024. The Google Scholar search produced more returns than the ProQuest; however, the relevance of the reports to DYMERS was lacking after the ninth return. From the tenth return onwards, the relevance to the topic decreases. Initially, the work was missing healthcare providers; then, the work was not a research study. Finally, the report was not peer-reviewed.

The final materials of this scoping review are two returns from Google Scholar.

## 3. Results

The results of the PRISMA process for the scoping review are found in Figure 1.

Table 1 provides the reports of the included studies.

The results of the included reports from the 9 December 2024 search of the various databases are the concern of this section. Two articles meet all the criteria; therefore, the subsections relate to each, appearing in the order returned in the search process. The first returned was the article published in April 2024, while the second was online at the end of December 2023. It was included in the first journal issue for 2024—providing its reference date.

### 3.1. DYMERS: A Working Hypothesis

“Dysregulation of mood, energy, and social rhythms syndrome (DYMERS): A working hypothesis” [55] is the first report returned that met all the inclusion criteria and none of the exclusion criteria for the search. Among the authors of this paper are two from the Department of Medical Sciences and Public Health, University of Cagliari, Italy—Carta and Primavera. This university, with Primavera in particular, has been the most productive regarding research into DYMERS [2,20,21,22,55,56,57].

The article relates that scoring positive on the Mood Disorder Questionnaire (MDQ) was closely associated with the dysregulation of sleep rhythms. A screening tool for bipolar disorder, the MDQ was considered unreliable because of the high number of false positives. However, noted by these authors is that regardless of whether they were diagnosed with bipolar disorder, those with a positive score invariably had a severe impairment in quality of life. Based on this observation, the identification was that three different types of hyperactivation can cause the positive test result produced from a dysregulation of behavioral rhythms. The first type represents a false positive caused by an increase in energy resulting from the type of high performance demonstrated by athletes. Continuous stress hormone stimulation results in the second type of false positive—the burnout experienced by medical professionals during COVID-19 is the example. The third positive is the hyperactivity that produces manic episodes in bipolar disorder. Dysregulation of Mood, Energy, and Social Rhythms Syndrome (DYMERS) is the working hypothesis accounting for the second level. Additionally, DYMERS includes that those at this second level are vulnerable to more pathological conditions that may evolve, such as bipolar disorder. DYMERS, then, is not only a diagnosis of identification—it foretells potential future pathology.

The hypothesis is that alterations in sleep–wake cycles may be a triggering factor for the onset of bipolar disorder. This possibility came to the attention of these researchers during the COVID-19 pandemic because of the dysregulation in social and behavioral rhythms resulting from lockdown requirements. Previously to the pandemic, regularity of rhythms was noted as a resilience factor. Regarding healthcare professionals, not leaving the hospital for weeks because of a fear of infecting family members while experiencing the effects of the pandemic on patients was instrumental in producing the hyperactivation of energy that led to burnout. This available energy increase raises blood pressure, improving physical and mental performance. Concomitantly, the resulting tension leads to sleep dysregulation—for some, adaptation results. For others with a predisposition to bipolar disorder, this can lead to continued hyperactivity, irritability, and debilitating disruptions to social and behavioral rhythms. The more the hyperactivation increases in this regard, the more burnout identifies the situation as unsolvable.

The authors end this report with suggestions for future research directions regarding this working hypothesis of DYMERS. Concerning biological rhythms and stress, they suggest (1) substantial sample studies to verify links with hyperactivation, dysregulation of biological rhythms, and stress, (2) comparisons with genetic vulnerability responses, and (3) studies of different personality profiles regarding approaches to stressful tasks.

### 3.2. Responses to Stressful Conditions as Evidence of DYMERS

“Does the Response to a Stressful Condition in Older Adults with Life Rhythm Dysregulations Provide Evidence of the Existence of the “Dysregulation of Mood, Energy, and Social Rhythms Syndrome”?” [56] is the second paper returned, but the first published of the two results. Primavera is the first author, with five of the other nine authors associated with the Department of Medical Sciences and Public Health, University of Cagliari, Cagliari, Italy. The aim is to investigate whether older adults with DYMERS had a more negative perception of their health-related quality of life during the COVID-19 pandemic lockdown.

One aspect of this study was regarding healthcare professionals, as rhythm dysregulation emerged as a burnout feature experienced by healthcare professionals under professional pandemic-related stress during COVID-19. The authors mention that a study analyzing the effects of night shift work on nurses’ mental well-being found that disruptions in circadian rhythms, along with poor sleep quality and quantity, significantly impacted the long-term mental health of these nurses [42].

The findings of this study were that DYMERS can reduce the quality of life comparable to that of severe chronic illnesses, including psychiatric conditions (such as major depressive disorder, obsessive–compulsive disorder, and post-traumatic stress disorder). In this regard, it ranks just behind a highly debilitating disease like multiple sclerosis.

## 4. Discussion

Invited to contribute to this Special Issue on Stress, Rhythms Dysregulation, and Bipolar Spectrum, the author decided to conduct a scoping review on what appeared to be the most interesting development in this area. In choosing to undertake this scoping review on DYMERS, the author was initially unaware that the two Special Issue Editors, Carta and Cossu, were collaborators in this research. These editors did not influence the choice of topic, nor did they know that their work would be the focus of this review before the submission. This statement clarifies that there is no conflict of interest in conducting this scoping review.

The author approached this undertaking from the perspective of a researcher who has developed a hospital-based offering for healthcare researchers who self-identify as experiencing burnout. There are several publications on the participant results, particularly regarding those who developed non-work-related depression and anxiety over the intervention [58,59,60,61,62]. A project that began in 2015 as the Health Narratives Research Group and continues, post-COVID-19, as the one-on-one Health Narratives Research Process, the author noted with the onset of the COVID-19 pandemic that a small percentage of participants developed more debilitating forms of depression and anxiety apart from their work-related burnout. When the author initially read about DYMERS being particularly affected by and identified during the COVID-19 pandemic, the explanation of the three levels of dysregulation and the potential causes of moving from the second level of burnout to expressing the depression and anxiety symptoms of bipolar disorder seemed to offer a perspective for what may have happened with the few participants who expressed more extreme, non-work-related symptoms in the course of participating in the offering during the pandemic. This potential explanatory power of DYMERS represents why the author undertook this scoping review.

The idea of a biopsychological emotion dysregulation framework regarding bipolar disorder originated before COVID-19, with the behavioral activation system dysregulation theory summarized in 1989 [63], and reviewed in 2008 [64]. However, this theory focuses predominantly on manic episodes of patients with bipolar disorder, with the development of depressive episodes less well explained [17]. Regarding the relationship between burnout and the non-work-related development of depression and anxiety during COVID-19, DYMERS appears as the only theory that can account for this effect, making it especially valuable for future investigations.

Two sites have investigated DYMERS—Cagliari, Italy, and Tunis, Tunisia—by interrelated research groups. The COVID-19 outbreak and its effect on those with bipolar disorder was the impetus for their research. Since the initial publication of these teams in 2021 [12], there have been nine additional publications—one from 2022 [11], three from 2023 [20,21,56], and five from 2024 [2,22,23,55,57]. Included in these reports are the two examined for this scoping review that relate particularly to burnout in healthcare professionals [55,56]. These publications continue to demonstrate and emphasize that the dysregulation caused by the COVID-19 lockdowns reduced the resilience of those in stressful situations, such as healthcare professionals, and was the impetus for their experience of burnout. With prolonged dysregulation in mood, energy, and social rhythms that healthcare professionals experienced during COVID-19, this dysregulation could then lead to the pathological depression and anxiety experienced by the least resilient, described as equivalent to a debilitating chronic disease [56].

### Limitations

The PRISMA extension for scoping reviews provides a recommended framework for methodological issues [65] followed for this scoping review. However, in using this framework, the charting process structure and synthesis control the information recorded [66]. As the charting process restricts the required information, the text offers additional details on the exclusion process, including the information in Table 1.

In selecting a scoping review as the method, the choice was not a systematic review with meta-analysis [48]. Compared with the systematic reviews in healthcare originating in the 1970s, scoping reviews are relatively new [67] and demand fewer detailed comparisons in their structured process. A scoping review aims to map the contextual breadth to identify existing evidence [68]. In not undertaking the critical appraisal of the methodological quality of the included studies, data extraction, analysis, and evidence applicability associated with a systematic review, and statistically estimating the data effect extracted from the individual studies through a meta-analysis, this scoping review is limited [69].

No guidelines require that scoping reviews be managed as a team [50,65,68]. Therefore, if completed by an independent researcher, a cognitive bias is possible [70]. Measures are necessary to overcome this possibility [71]. The author has provided a detailed description of the searches performed in the Section 2 and included in Table 1, providing the databases of the studies returned for each search conducted to mitigate cognitive bias. Creating a detailed color-coded system identifies and differentiates articles following the PRISMA process, permitting the reproduction of this study. By making the decisions regarding each of the 18 studies considered for inclusion available for inspection and by including the Preferred Reporting Items for Systematic Reviews and Meta-Analyses Extension for Scoping Reviews (PRISMA-ScR) Checklist as **Supplementary S1**, the author actively intends to alleviate cognitive bias.

As a topic that came to the attention of researchers only resulting from the lockdowns associated with COVID-19, the few relevant returns from the database searches—and that it was Google Scholar that returned these relevant reports rather than one of the primary databases—define this review [49,53]. This limitation is further exacerbated by, to date, only one two-site research group conducting investigations and publishing on this topic. However, this limitation does not represent a selection bias [72] as there is no other research on this topic—it is new and currently limited to two research sites. This scoping review is not a systematic review and meta-analysis. Therefore, there was no statistical analysis, providing further reason why a selection bias was not evident [73]. That there are only two relevant returns for this scoping review does not undermine the quality of this work. Although scoping reviews are intended to produce a wide range of results, if these results are not available because of the newness of the topic investigated this is not dismissive of the scoping review process. According to the most recent research on recommendations for scoping reviews from 2023 [74], it is the PRISMA process that determines a scoping review, not the quantity of results.

By investigating the range of publications available on DYMERS, this scoping review did not consider physiological factors regarding burnout in healthcare professionals. Burnout involves both psychological and physiological responses to environmental stressors such that heart rate variability is a non-invasive marker indicating the functioning of the autonomic nervous system regarding burnout [74]. Lower heart rate variability among healthcare professionals reporting COVID-19 stress and burnout has been identified [75]. Furthermore, saliva cortisol and blood concentration of glycated hemoglobin are significantly associated with emotional exhaustion in physicians, recognizing burnout [76]. It is a limitation that this study focused only on psychological and social considerations particularly related to a diagnosis of DYMERS.

## 5. Conclusions

Dysregulation of Mood, Energy, and Social Rhythms Syndrome (DYMERS) is a new syndrome identified during COVID-19 to account for the Mood Disorder Questionnaire (MDQ), recognizing a larger number of false positives compared to diagnoses of bipolar disorder. In accounting for these positives (false and otherwise), DYMERS is a heuristic particularly associated with the second of three levels. The first level was the increase in energy demonstrated by high-performance athletes, while medical professional burnout during COVID-19 identified the second level. These levels represent the false positives of the MDQ. The third correctly recognizes manic episodes in bipolar disorder.

This scoping review examines the range of publications on DYMERS regarding burnout in healthcare professionals. The criteria included two reports. They point to the severe level of disability that can result from DYMERS, calling for sample studies with a large population to verify its links with hyperactivation, dysregulation of rhythms, and stress, and comparisons with genetic vulnerability responses and personality profiles in approaching stressful tasks. As this is research by a limited number of scientists associated with the same team on only two sites, future research should extend to more teams, a greater geographic area, and more diverse samples of healthcare professionals. The call is for this research to begin quickly, as the effects of the COVID-19 lockdowns in this regard are waning. Such research on DYMERS will then be relevant concerning subsequent pandemics. With the resulting research, practical interventions for healthcare professionals regarding the identification of DYMERS would then be advisable to reduce the level of potential disability. What these interventions might be would depend on the outcome of such research. However, with a diagnosis of DYMERS along with burnout, a focus of practical interventions could be improving sleep and sleep patterns in healthcare professionals previously neglected before the onset of COVID-19.

## Figures and Tables

**Table 1:** Numbered 9 December 2024 results of database searches of “Stress Rhythms Dysregulation Bipolar Disorder Burnout DYMERS Healthcare Professionals COVID-19” including article title, database returning the report, year of publication, journal, authors, and whether the report is excluded or has a citation number—excluded reports are color-coded.

#	Title	Database	Year	Journal	Authors	Excluded or Citation #
1	Hyperactivity and Risk for Dysregulation of Mood, Energy, and Social Rhythms Syndrome (DYMERS): Standardization of a Simple One-Item Screener versus the Mood Disorder Questionnaire (MDQ)	ProQuest	2024	Journal of Clinical Medicine	Ouali U, Aissa A, Reiaibi S. Zoghlami N, Larnaout A. et al., Primavera D.	Duplicated with Google Scholar
2	Are Depressive Symptoms in Obstructive Sleep Apnea Attributable to a Syndrome of Dysregulation of Rhythms and Hyperactivity (DYMERS)?	ProQuest	2024	Journal of Clinical Medicine	Primavera D, Cantone E, Cannizzaro GM, Sanna C, Redolfi S.	Duplicated with Google Scholar
3	Cognitive Impairment and Risk of Depressive Episodes from a Bipolar Spectrum Perspective: A Case-Control Study in Older Adults during the COVID-19 Lockdown	ProQuest	2024	Psychiatry International	Primavera D, Fabrizio B, Romano F, La Torre G, Gonzales A, Ivan C, et al.	
4	Examining Anxiety and Insomnia in Internship Students and Their Association with Internet Gaming Disorder	ProQuest	2024	Journal of Clinical Medicine	Alshammari TK, Rogowska AM, Alobaid AM, Alharthi NW, Albaker AB, et al.	Duplicated with Google Scholar
5	Dysregulation of mood, energy, and social rhythms syndrome (DYMERS): A working hypothesis	Google Scholar	2024	Journal of Public Health Research	Carta MG, Fornaro M, Primavera D, et al.	[55]
6	Does the Response to a Stressful Condition in Older Adults with Life Rhythm Dysregulations Provide Evidence of the Existence of the “Dysregulation of Mood, Energy, and Social Rhythms Syndrome”?	Google Scholar	2023	Healthcare	Primavera D, Aviles Gonzonlez CI, Romano F, Kalcey G, et al.	[56]
7	Hyperactivity and Risk for Dysregulation of Mood, Energy, and Social Rhythms Syndrome (DYMERS): Standardization of a Simple One-Item Screener versus the Mood Disorder Questionnaire (MDQ)	Google Scholar	2024	Journal of Clinical Medicine	Ouali U, Aissa A, Reiaibi S. Soghlami N, Larnaout A. et al., Primavera D.	
8	Are Depressive Symptoms in Obstructive Sleep Apnea Attributable to a Syndrome of Dysregulation of Rhythms and Hyperactivity (DYMERS)?	Google Scholar	2024	Journal of Clinical Medicine	Primavera D, Cantone E, Cannizzaro GM, Sanna C, Redolfi S.	
9	Stress, Dysregulation of Rhythms, and Bipolar Disorder: A Challenging Field of Research	Google Scholar	2024	Journal of Clinical Medicine	Carta MG, Karam EG, Cossu G, et al.	
10	Cognitive Impairment and Risk of Depressive Episodes from a Bipolar Spectrum Perspective: A Case-Control Study in Older Adults during the COVID-19 Lockdown	Google Scholar	2024	Psychiatry International	Primavera D, Bert F, Romano F, La Torre G, et al.	
11	Examining Anxiety and Insomnia+B12 in Internship Students and Their Association with Internet Gaming Disorder	Google Scholar	2024	Journal of Clinical Medicine	Alshammari TK, Rogowska AM, Alobaid AM, Alharthi NW, Albaker AB, et al.	
12	Mental Disorders and DNA Methylation	Google Scholar	2024	Handbook of the Biology and Pathology of Mental Disorders	Orenay- Boyacioglu S, Boyacioglu O, et al.	
13	The Current Status of Cognitive Disorders and Their Diagnosis	Google Scholar	2024	Cognitive Predictive Maintenance Tools for Brain Diseases: Design and Analysis	Dev P, Pathak A et al.	
14	The Current Status of Cognitive Disorders and Their Diagnosis	Google Scholar	2024	Cognitive Predictive Maintenance Tools for Brain Diseases: Design and Analysis eases	Dev P, Pathak A et al.	Duplicated with Google Scholar
15	Sleep Hormone Melatonin, Inflammation and Aging	Google Scholar	2023	Sleep and Clocks in Aging and Longevity	Xia Y, Wu X, Yin Z, Li Y, He F, et al.	
16	Targeting Mitochondria 2023 Abstract Book	Google Scholar	2023	Journal of Mitochondria, Plastids and Endosymbiosis	Weeissig V, Edeas M	
17	Diet, metabolism and cancer progression	Google Scholar	2023	FEBS Open Bio	FSHK Lecture-Opening	
18	Abstracts presented at the 25th Annual Congress of the Belgian Society of Internal Medicine, 10-11 December 2021, Dolce La Hulpe, La Hulpe, Belgium	Google Scholar	2021	International Journal of Clinical and Laboratory Medicine	Van Dorpe I, Acta Clinica Belgica	

Color legend (order of exclusion appearance). Yellow: duplicate; gray: irrelevant information on healthcare professionals; dark gray: no mention of healthcare professionals; green: not a research study; red: not peer reviewed.

## Supplementary Materials

The following supporting information can be downloaded at https://www.mdpi.com/article/10.3390/jcm14031035/s1. Supplementary S1. Preferred Reporting Items for Systematic Reviews and Meta-Analyses Extension for Scoping Reviews (PRISMA-ScR) Checklist.
